# Intake of Nutritional Supplements among People Exercising in Gyms in Beirut City

**DOI:** 10.1155/2012/703490

**Published:** 2012-02-01

**Authors:** D. El Khoury, S. Antoine-Jonville

**Affiliations:** ^1^Department of Nutritional Sciences, Faculty of Medicine, University of Toronto, Toronto, ON, Canada M5S 3E2; ^2^Department of Nutrition and Food Sciences, Faculty of Agricultural and Food Sciences, American University of Beirut, Beirut 1107 2020, Lebanon; ^3^Faculty of Sports Sciences, EA3596, University of French West Indies, Pointe-à-Pitre 97157, France

## Abstract

The use of nutritional supplements among exercisers in gyms has been never investigated in the Middle East. The aim of the current study was to assess the prevalence intake of nutritional supplements and the potential influencing factors among people exercising in gyms in Beirut city. In this cross-sectional study, 512 exercisers, aged between 20 and 50 years, were randomly selected from gyms. The intake of nutritional supplements was reported among 36.3% (95% confidence interval 32.2–40.5) of participants, with a weak presence of medical supervision. Patterns of supplement use differed by gender and age. Men and younger exercisers were found to focus on supplements associated with performance enhancement and muscle building, while women and older exercisers were more concerned with health-promoting products such as vitamins, minerals, and herbal supplements. An appropriate dissemination of accurate and scientifically sound information regarding the benefits and side effects of nutritional supplements is highly recommended in the sports environment in Beirut city.

## 1. Introduction

Lebanon is a Mediterranean country with an estimated population of about 4 million, of which 40% lives in the capital Beirut. Lebanon has been regarded as a country in transition, experiencing a shift in disease type and prevalence as well as in lifestyle and dietary habits [1]. The consumption of nutritional supplements is a major dietary component that might have affected the Lebanese culture through westernization and urbanization.

Nutritional supplements are food components, for example protein, and not foods, or pharmaceutical preparations, for example vitamins and minerals capsule or tablet, supplying one or more nutrients in a concentrated form including proteins, minerals, vitamins, trace elements, and other components that are theoretically present in a normal and balanced diet [2]. Nutritional supplements are usually offered in an untypical form of food, including tablets, capsules, powders, or pills. Although many individuals use supplements, those engaged in sport and physical activities were found to represent an important portion of people purchasing supplements [3].

There is enough evidence that physically active people do not require additional nutrients apart from those obtained from a balanced diet [4]. The American Dietetic Association, the Dietitians of Canada, and the American College of Sports Medicine stated that only those persons who restrict their energy intake, use severe weight-loss practices, eliminate one or more food groups from their diets or consume high-carbohydrate diets with low micronutrient density may require dietary supplementation [5].

Despite these recommendations, the use of supplements has greatly increased in the past years [6]. In fact, nutritional supplements' industry is currently an international market worth billions of dollars [7]. In the United States, more than 3 million people were reported to be using or to have used ergogenic supplements [8]. There has been an increasing use of nutritional supplements by people engaged in physical or athletic activities [9]. Supplement use among athletes is well documented. It was estimated to range from 40 to as high as 88% [10, 11] and was found to vary according to types of sports, cultural aspects, age groups, and gender [3].

Apart from elite sports club, the highest density of supplement users may reside in the gyms. People exercising in gyms may represent a major target for the supplement market due to their increased widespread existence and to the ease of their access to a variety of sports foods and nutritional supplements. However, limited number of studies described the prevalence of supplement use among people exercising in gyms [12–14].

The increase in demand for nutritional supplements was attributed to various reasons, including enhancing performance, improving health, preventing nutritional deficiencies and illness, increasing muscle mass, decreasing body fat, boosting immunity, increasing alertness and mental activity, improving recovery, and reducing stress [15–18]. However, the exact benefits of nutritional supplements are still not well established [5, 19, 20]. Moreover, various potential risks were described when using nutritional supplements in high doses or without medical supervision [21].

The practices of athletes and exercising individuals in the Middle East region, and more specifically in Lebanon, remain undocumented. Thus, this study aimed to assess the prevalence of nutritional supplements' intake among people who exercise in gyms in the city of Beirut, and to discuss the potential influencing factors as well as the major sources of information and motives associated with their use in an effort to tailor the adequate nutritional awareness campaigns targeted towards exercisers themselves as well as people influencing their decisions.

## 2. Materials and Methods

This was a cross-sectional study carried out in the city of Beirut, Lebanon, from June 2010 to August 2010.

### 2.1. Selection of Population

The sample size calculation (*n* = 504) was based on the equation *n* = *z* ^2^∗*p*∗(1 − *p*)/*W* ^2^, where *n* was the estimated sample size, *z* the normal distribution (defined as 1.96 for research with 95% confidence), *p *the estimated proportion of individuals who use supplements (30%), and *W* the study margin of error (4%). A total of 512 participants were included in the study.

A two-stage sampling was performed. In the first stage, all commercial gyms were identified based on the address listings provided by various public and information agencies in the city of Beirut. Gyms that reported offering only one type of physical activity were excluded from the study. Telephone contacts were carried out in order to assess their current existence and activities. In the second phase, a systematic random scheme was used in order to select adults from the eligible gyms (*n* = 50) in proportion to the population size of each district of Beirut city, including Achrafieyh, Ain el Maryseh, Bashoura, Mazraa, Minet el Hosn, Mousaytbeh, Ras Beirut, Rmeil, Saife, Zkak el Blat, Port, and Dahieh. Such selection allowed for a more regular distribution of participants from various socioeconomic groups. To be included in the study, individuals had to be exercisers aged between 20 and 50 years with no regard to gender or socioeconomic status. All participants signed a formal consent after being informed about the objectives of the study.

The study was conducted according to the principles expressed in the Helsinki Declaration and was approved by the Institutional Review Board of the American University of Beirut, Lebanon.

### 2.2. Questionnaire

The administered questionnaire consisted of 17 questions, divided into three main parts. The first part included questions concerning demographic characteristics such as age, gender, educational background, disease history, alcohol consumption, and smoking status. The second part tackled sports-related features, such as type, frequency, and total duration of physical activities. The third part of the questionnaire consisted of questions related to supplement use. In this section of the questionnaire, participants had to mention about the sources of sports nutrition information, the motivations for the use of nutritional supplements, as well as the types of supplements being used and the duration and timing of their consumption. This questionnaire was previously tested in a pilot study conducted in gyms from two different regions of the city of Beirut, and a final version was developed. All surveys were filled by the researchers themselves, after reading the questions to the participants.

### 2.3. Statistical Analysis

Statistical analyses were conducted using SPSS software (version 17.0; SPSS, Chicago, Ill).

All variables were categorical. Descriptive analyses were based on frequencies and percentages. Pearson chi-square tests were used to identify associations of supplement intake status (user/nonuser) with factors potentially related to its use.

Independent two-step cluster analyses were used to motivate modality grouping within each question (types of nutritional supplements, reasons for supplement use, and sources of supplement information). This method creates groups of subjects as homogeneous as possible inside each group, and as contrasted as possible between groups. It combines sequential and hierarchical agglomerative methods preclustering and then subclustering data. The determination of the number of clusters was based on the largest relative increase in distance between the two closest clusters defined by the Schwarz Bayesian Criterion as well as on potentially meaningful explanations. The type of nutritional supplements in use was best described by the following modalities: performance supplements (aggregating creatine, amino acid pills, arginine, glutamine, and branched-chain amino acids), vitamins/minerals (including multivitamins, multiminerals, vitamin and mineral supplements, as well as vitamin C and vitamin E), weight/fat loss supplements (including carnitine, protein bars, and protein shakes), alertness/energy supplements (comprising caffeine and sports energy drinks), natural supplements (with herbal supplements, iron, calcium tablets, and fish oil pills), and the last cluster included soy and sports bar. Finally, protein powder, casein protein, whey protein, and antioxidants remained independent as they were not found to fit into any of the six clusters. Four clusters have been identified covering all reasons for supplement use among those proposed: disease prevention (prevention of nutritional deficiencies, treatment of medical problems, and prevention of diseases in the future), immunity/energy boosting (immunity boosting, increased alertness and mental activity, and decreased stress), muscle building (muscle gain or weight gain, muscle repair or recovery, strength enhancement, and performance improvement), and weight/fat loss (meal replacement, weight loss, and decreased body fat). The sources of supplement information were described by three clusters: medical/paramedical (dietitians and physicians), media (magazines, internet, media, books, and friends), and coaches (coaches and companies). These clusters have been used for subsequent analyses except otherwise stated.

Types of nutritional supplements, reasons for supplement use, and sources of supplement information were analyzed by gender and age group. Several variables were found to influence these nutritional supplements-associated parameters. Age appeared as a common variable in few studies [22, 23]. On the other hand, the role of gender is still not clear [10, 24]. The odd ratio measured the associations of types of nutritional supplements, reasons for supplement use, and sources of supplement information with male and female gender from one side and various age groups from another side.

Differences were considered statistically significant at *P* < 0.05.

## 3. Results

### 3.1. Demographic Characteristics

Table 1 summarizes the basic characteristics of participants. Out of the 512 participants, 60.9% were men. The population was young, with 63.7% being in the 20–30 year age group. More than half of exercisers (69.1%) reported having a university degree, including bachelor degree, masters degree, and above.

### 3.2. Alcohol Consumption, Smoking and Disease History

As shown in Table 1, 65.6% were nonconsumers of alcoholic beverages. In addition, 61.7% were nonsmokers, and 92.8% described no disease history at the time of the survey.

### 3.3. Physical Activity

Most of those interviewed exercised for more than a year (75.4%) (Table 1). The majority of participants exercised regularly for 3 to 5 times a week (64.1%) and 68.6% spent 1 to 2 hours per day exercising. The highest percentage of exercisers mainly performed strength training (65.4%) and treadmill (63.5%) in gyms (data not shown).

### 3.4. Use of Nutritional Supplements

The intake of nutritional supplements was reported by 36.3% (95% confidence interval 32.2–40.5) of participants.

Supplement use status was significantly associated with gender and total time of exercise (Table 1). Among users, a large proportion were men (72.0%, *P* < 0.001) and were participants who have been exercising for a duration longer than a year (83.3%, *P* < 0.01). Other factors, including age group, education, alcohol intake, smoking status, disease history, frequency of exercise, and total time of daily exercise, had no significant associations with supplement use.

When analyzing supplement use by types of physical activities in Table 2, only strength training (*P* < 0.001), treadmill (*P* < 0.05), and fights and martial arts (*P* < 0.05) had significant associations with the use of nutritional supplements. While exercisers performing strength training (80.6%) and treadmill (57.0%) reported significantly higher percentages of supplement use as compared with exercisers involved in other activities, those performing fights and martial arts had significantly lower levels of intake of nutritional supplements (14.5%).

### 3.5. Types of Supplements

**Figure 1:**
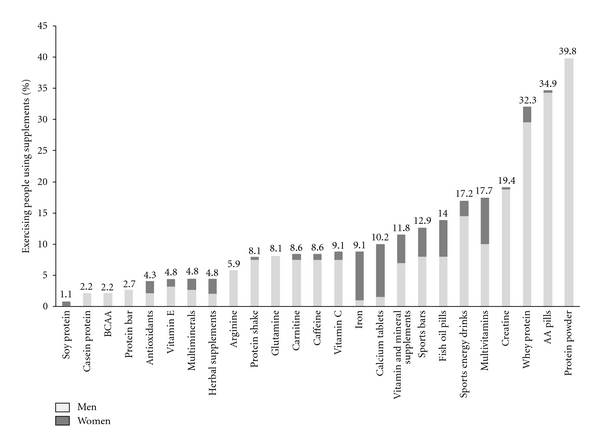
Types of nutritional supplements used among supplement users (*n* = 186); BCAA: branched-chain amino acids; AA pills: amino acid pills. There are no missing data.

The types of nutritional supplements consumed by participants are shown in Figure 1. The five most commonly consumed supplements were protein powder (39.8%), amino acid pills (34.9%), whey protein (32.3%), creatine (19.4%), and multivitamins (17.7%). The intake of two or more products simultaneously was reported by 25.6% of participants (data not shown).

 Almost half of the participants (44.1%) reported using supplements for more than 2 years, while 22% described taking them for 1 to 2 years (data not shown).

#### 3.5.1. Types of Supplements according to Gender

As shown in Table 3, men consumed performance supplements (*P* < 0.001), weight/fat loss supplements (*P* < 0.05), alertness/energy supplements (*P* < 0.05), protein powder (*P* < 0.001), and whey protein (*P* < 0.001) to a greater extent than women. On the other hand, women focused more on products associated with health benefits, including vitamins/minerals (*P* < 0.01) and natural supplements (*P* < 0.001) in comparison to men.

#### 3.5.2. Types of Supplements according to Age Group

Types of used supplements differed as well by age groups as shown in Table 3. Participants aged above 40 years consumed less of the performance supplements (*P* < 0.01) and protein powder (*P* < 0.05) in comparison to those aged below 30 years. Moreover, exercisers aged between 30 and 40 years were found to take weight/fat loss supplements to a lesser extent than those younger than 30 years (*P* < 0.05). On the other hand, older exercisers consumed more of the natural supplements in comparison to younger ones (*P* < 0.001).

### 3.6. Reasons for Supplement Use

**Figure 2:**
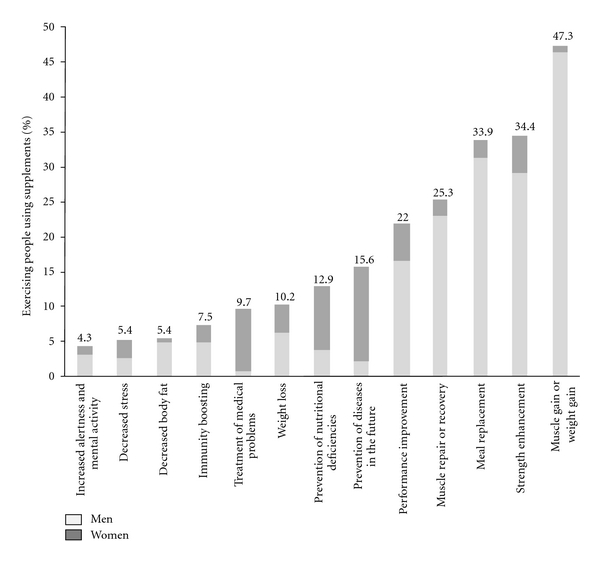
Reasons for supplement use among supplement users (*n* = 186). There are no missing data.

The major reported reasons for supplement use were to promote muscle gain (47.3%) and to enhance strength (34.4%, Figure 2). Other reasons included to replace meal (33.9%), increase muscle repair or recovery (25.3%), and enhance performance (22.0%).

#### 3.6.1. Reasons for Supplement Use according to Gender

As shown in Table 4, men focused on the muscle building (*P* < 0.001) and fat reduction (*P* = 0.001) aspects of nutritional supplements to a greater extent than women. On the other hand, women were more interested than men in the health-related aspects of supplements, such as disease prevention (*P* < 0.001).

#### 3.6.2. Reasons for Supplement Use according to Age Group

When analyzing the reasons for using nutritional supplements by age group, significant differences were also noted (Table 4). Exercisers aged between 30 and 40 years were less concerned about the muscle-building functions of supplements in comparison to younger ones (*P* = 0.050). Participants older than 40 years were even less concerned (*P* < 0.01). On the other hand, exercisers above the age of 40 years had an increased interest in the disease prevention characteristics of nutritional supplements in comparison to those younger than 30 years (*P* < 0.001).

### 3.7. Sources of Supplement Information

**Figure 3:**
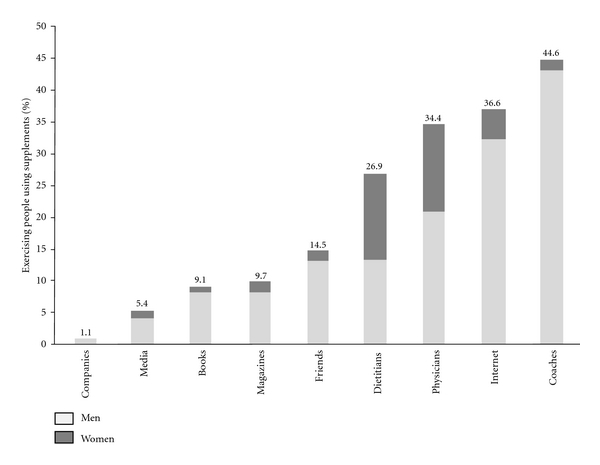
Sources of information among supplement users (*n* = 186). There are no missing data.

A big proportion of participants consumed supplements without seeking any professional guidance (Figure 3). The highest percentage of supplement users was found to seek information from “uncertain” sources, including coaches (44.6%) and internet (36.6%). Media, including magazines, internet, media, and books, constituted an important source of supplement information for exercisers. In fact, 60.8% of supplement users were found to rely on media-related sources. On the other hand, only 34.4% and 26.9% of exercisers got information from medical sources including physicians and dietitians, respectively.

#### 3.7.1. Source of Supplement Information according to Gender

Sources of information regarding nutritional supplements were found to differ among males and females (Table 5). Men relied mainly on nonmedical sources, including media (*P* < 0.001) and coaches (*P* < 0.001). On the other hand, women cared more to seek information from medical professional sources including dietitians and physicians (*P* < 0.001).

#### 3.7.2. Sources of Supplement Information according to Age Group

When analyzing data according to age group, a trend was also observed (Table 5). Younger exercisers, aged below 30 years, got their information regarding nutritional supplements from coaches to a significantly greater extent than those aged above 40 years (*P* < 0.01). On the other hand, older exercisers, aged above 40 years, consulted with medical/paramedical professionals to a greater extent than younger age groups (*P* < 0.01).

### 3.8. Supplement Labels

A rate of 87.6% of supplement users reported checking labels on nutritional supplements (data not shown). Amongst those who indicated not reading labels, almost half of them (52.2%) were found to trust enough their trainers.

## 4. Discussion

Our study is the first to illustrate the prevalence use of nutritional supplements among exercisers, not athletes, in gyms in the Middle Eastern region. In the current study, 36.3% of participants reported using nutritional supplements among a representative sample of people exercising at gyms in Beirut city. Such prevalence rate is almost the same as that described in the study of Goston and Correia [13] (36.8%) among exercisers in gyms in the city of Belo Horizonte, Brazil. Even if significant, supplement use in Beirut city was found to be way lower than rates observed in New York City (84.7%) and in Spain (56.1%) among exercisers [12, 14]. Exercisers in gyms constitute an important target for nutritional supplement market. However, most studies investigating about supplement use worldwide focused on athletes [10, 15, 18, 25–27], among which the highest percent of consumers is found [28]. Discrepancies in reported prevalence rates could be explained by the different types of gyms included in the studies, the varied characteristics of participants, the various modes of data collection, as well as the potential under- or overreporting of supplement use and the lack of knowledge about the exact definition of supplement among exercisers [16, 24, 29, 30]. In the current study, only gyms offering different sports activities were selected in order to avoid the inclusion of people focusing on body-building activities. It is well established that body-builders consume more nutritional supplements than other sport participants [10, 27].

Supplement use patterns were found to differ by gender and total time of exercise. The role of gender as a determinant of supplement use is still not clearly established. In the current study, male exercisers constituted a bigger proportion of supplement users taking into account that more males participated in the study. Similarly, supplement use was reported to be higher among male exercisers [13] and adolescent athletes [31]. In contrary, Sobal and Marquart [10] found a significantly higher prevalence of supplement use among female athletes. On the other hand, Sundgot-Borgen et al. [24] did not find any significant difference between sexes. The association of exercising duration with the status of supplement use has been rarely investigated. In the current study, participants who have been exercising for a long duration were the ones to consume nutritional supplements the most. A greater exercising period is most likely associated with increased interest in sports-related information, increased exposure to sports-related media, and increased interaction with trainers, coaches, and sports fellows. All these factors were described with major impacts on the decision-making process regarding the use of nutritional supplements [32]. At last, differences in supplement use patterns can be also observed between various sports activities. Supplement use is most frequent in sports that emphasize performance and muscle size. In fact, consumption of nutritional supplements in strength sports such as weight lifting, power lifting, and bodybuilding was found to be higher than that in other sports [10, 33]. In the current study, strength training, and to a lesser extent treadmill, was associated with a greater utilization of nutritional supplements, unlike fights and martial arts that do not usually emphasize on muscle building and strength.

Similarly to the current study, protein supplements were among the most widely used nutritional ergogenic supplements in other studies, ranging from 28% in Sevilla, Spain [14], and 42.3% in New York City [12], up to 58% in Belo Horizonte, Brazil [13]. These observations originate primarily from misconceptions regarding protein supplement effectiveness [34]. However, the association between protein intake and muscle mass has not been yet scientifically proven [34, 35]. Even in athletes involved in intense training and with elevated dietary protein needs, a well-balanced diet that maintains energy balance can achieve these recommendations [36–38]. On the other hand, as observed in the current study, vitamin and mineral supplements were found to be frequently consumed in exercise [12–14]. Although physical activity may slightly increase the requirements for certain vitamins and minerals [39, 40], many of which are involved in muscle contraction as well as in energy store replenishment and muscle repair, this is generally met by the high energy intake of exercisers and athletes [39].

In the current study, men and younger exercisers focused on supplements generally associated with performance enhancement, body building, and fat reduction including those rich in proteins and amino acids, while women and older exercisers were more concerned with supplements with general health benefits such as those rich in vitamins, minerals, and herbal products. Such pattern has been similarly observed in various studies [12, 15, 16, 41, 42]. In the study of Goston and Correia [13], men and young exercisers were more likely to use supplements rich in proteins and branched-chain amino acids while women and persons older than 40 years took supplements rich in vitamins/minerals and natural/phytotherapeutic agents. Similarly, female varsity college athletes were more likely to take calcium and multivitamins, while males had a significantly important intake of amino acids, glutamine, weight gainers, and whey protein [16].

Reasons for selecting nutritional supplements were various, differing as well according to gender and age group. In general, females were more likely to take supplements for health or because of inadequacy in dietary habits, while males reported taking supplements to improve speed, strength, power, and weight/muscle gain [10, 15, 16, 25, 30, 43, 44]. In the present study, supplement use was essentially revolving around muscle gain, muscle repair, and performance enhancement in men and young exercisers. On the other hand, women and old exercisers used nutritional supplements for health-oriented purposes, including prevention of nutritional deficiencies, treatment of medical problems, and prevention of future diseases. Similarly, in the study of Goston and Correia [13], people younger than 30 years took supplements with the purpose of increasing muscle mass, while those older than 45 years consumed supplements to prevent future illness. Moreover, the use of multivitamins, vitamin C, and herbal products was higher among female university athletes in Singapore for general health benefits, while athletes who wanted to gain muscle mass used protein and amino acids [45]. Among exercisers in New York City, more of the oldest participants consumed multivitamins, minerals, and vitamin E to prevent future illness, while those aged 45 years or younger chose protein shakes and bars to build muscle [12].

The increased supplement utilization rates among exercisers could be partially attributed to a positive attitude towards supplements as a part of an appropriate dietary pattern [32, 46]. However, there is still no conclusive evidence that supplementation enhances health or sports performance. Supplementation may benefit athletes with preexisting deficiencies or who are on caloric restrictions or traveling for prolonged periods to regions with inadequate or limited food supply [39]. Most of supplement users are not familiar with the negative impacts of nutritional supplements on health if inappropriately used. In the study of Tian et al. [45] on university athletes in Singapore, 86.4% were unaware that supplements can adversely affect health. The potential risks of several nonanabolic nutritional supplements when used in high doses or without the counseling of a physician have been described [21]. In the current study, even if the consumption amounts of nutritional supplements were not assessed, such a high consumption rate may reflect inappropriate and dangerous consumption patterns.

This lack of awareness is majorly attributed to the inadequate sources of information of exercisers and athletes regarding nutritional supplements. Nearly 80% of athletes obtain information from “questionable” sources including media, internet, peers, coaches, and trainers [15, 17, 44, 47]. In the current study, coaches were the primary source of supplement information, mainly for younger male exercisers. Athletic trainers and coaches were also described as the most trusted professionals for supplement information by male athletes in other studies [11, 26, 48]. Advices provided by coaches and athletic trainers are usually inaccurate, inappropriate, or even potentially damaging. A big number of athletic trainers and coaches have a minimal specialized knowledge in sports nutrition. Moreover, they have ready access to and financial interest in an ever-increasing range of supplements and sports foods, which might greatly influence the type and direction of their consultations.

Media, including books, magazines, television, and internet, was also perceived as a powerful influence on a person's decision to use nutritional supplements [32]. Media, including internet, media, magazines, and books, influenced the decisionmaking of 60.8% of participants in the current study. Magazines, newspapers, and internet were also the major sources of information about supplements of 39.7% of university athletes in Singapore [45]. Similarly, internet (79%), magazines (68%), and television (52%) were the most popular media sources for supplement information of college athletes [48]. Exercisers are constantly exposed to advertisements in magazines and internet, which distort clinical studies and formulate misleading claims [31, 32, 49, 50]. Although media information may not be clinically validated [51], 52% of consumers were found to believe that “almost all” or “most” health information on the internet is credible according to the Pew Internet and American Life Project Report [52].

Consultation with medical professionals, including physicians and dietitians, is practiced to a lower extent in the sports environment. In the current study, 73.1% of exercisers had never received any guidance from a nutritionist. Similarly, Rocha and Pereira [53] showed that most athletes (78%) do not obtain their supplement information from nutritionists. In other studies, only 10 to 14% of participants considered dietitians as their primary source of supplement information [26, 44]. This observation could be majorly attributed to the limited access to health professionals, including dietitians, in gyms and sports centers.

Due to the limited knowledge about supplement safety and toxicity, polypharmacy, or the concurrent use of more than one product, is another major issue among athletes and exercisers [17, 43]. The highest rates were described among athletes. Baylis et al. [54] reported that 77% of elite Australian swimmers concurrently used more than one vitamin/mineral product. In a study on intercollegiate student athletes, 88% used one or more nutritional supplements [26]. Multivitamins were often combined with vitamin C, calcium, vitamin E, and iron by Singaporean university athletes [45]. Among exercisers, it was also commonly practiced though at lower rates. In the study of Goston and Correia [13], 43.5% of exercisers reported the simultaneous use of two or more products. In the current study, the rate of utilization of two or more products was almost half (25.6%). However, it remains significant and worth followup and intervention.

Product labeling was enacted in the United States by the Dietary Supplement Health and Education Act of 1994 in order to protect consumer access to nutritional supplements while providing guidelines for their consumption [51]. In the current study, 87.6% of supplement users did check supplement labels before consumption. This value is much higher and promising than those previously reported, where 33.3% [17] and 57% [15] of athletes attempted to seek information and read labels before using any supplement. However, if not properly supervised, reading labels might be erroneous and misleading. Both U.S. Food and Drug Administration and the American College of Sports Medicine encourage individuals to examine supplement safety, efficacy, potency, and legality prior to use under the supervision of healthcare professionals [40].

## 5. Conclusion

In conclusion, we identify a potential overintake of nutritional supplements among exercisers in the gyms of Beirut city. This pattern reflects a serious public health concern since practiced with minimal professional guidance. As nutritional supplements do not compensate for poor food choices and inadequate dietary habits and as they are not risk-free, it is extremely essential to disseminate accurate and scientifically sound information regarding appropriate use, potential benefits, and possible side effects of these products in the sports environment. Health professionals, including physicians, dietitians, and pharmacists, should combine their expertise with that of coaches and athletic trainers in order to provide more comprehensive nutrition services to exercisers.

## Figures and Tables

**Table 1:** Sociodemographic and lifestyle characteristics of all participants and of users of nutritional supplements. No missing data.

	Percentage of total population (*n* = 512)	Percentage of supplement users (*n* = 186)	*P *
Gender			
Male	60.9	72.0	<0.001
Female	39.1	28.0	

Age group (years)			
20–30	63.7	64.0	0.936
30–40	26.6	26.9	
40–50	9.8	9.1	

Education			
Brevet	10.2	9.1	0.615
Lebanese Bachelor	13.7	10.8	
Technical degree	5.5	6.5	
Bachelor	56.8	59.1	
Masters or above	12.3	12.4	
Others	1.6	2.2	

Alcohol intake			
No	65.6	60.8	0.080
Yes	34.4	39.2	

Smoking status			
No	61.7	59.1	0.500
Current	31.3	34.4	
Former	7.0	6.5	

Disease history			
No	92.8	90.3	0.106
Yes	7.2	9.7	

Total time of exercise			
<1 month	3.9	1.6	0.005
1–6 months	11.5	10.2	
7 months–1 year	9.2	4.8	
>1 year	75.4	83.3	

Frequency of exercise			
<3 times/week	16.8	14.0	0.099
3–5 times/week	64.1	62.4	
>5 times/week	19.1	23.7	

Total time of daily exercise			
<1 hour/day	18.0	16.7	0.673
1-2 hours/day	68.6	71.0	
>2 hours/day	13.5	12.4	

**Table 2:** Types of physical activities performed in the gyms by supplement users (*n* = 186). No missing data.

Types of physical activities	Percentage supplement use (*n*)	*P *
Strength training		
No	19.4 (36)	<0.001
Yes	80.6 (150)	

Treadmill		
No	43.0 (80)	0.021
Yes	57.0 (106)	

Team sport		
No	72.6 (135)	0.265
Yes	27.4 (51)	

Water sport		
No	80.6 (150)	0.808
Yes	19.4 (36)	

Fights and Martial Arts		
No	85.5 (159)	0.019
Yes	14.5 (27)	

Yoga		
No	94.1 (175)	0.515
Yes	5.9 (11)	

Others		
No	89.8 (167)	0.389
Yes	10.2 (19)	

**Table 3:** Types of nutritional supplements by gender and age group (*n* = 186). CI: confidence interval. No missing data.

Types of nutritional supplements	Gender			Age group (years)	
	Men	Women	20-30	30-40	40-50
Performance supplements cluster					
Percentage (*n*)	54.5 (73)	1.9 (1)	42.0 (50)	46.0 (23)	5.9 (1)
Odd ratio (95% CI)	1.000	0.016 (0.002-0.122)	1.000	1.176 (0.605-2.285)	0.086 (0.011-0.672)
*P *	<0.001		0.010		

Vitamins/minerals cluster					
Percentage (*n*)	23.9 (32)	46.2 (24)	28.6 (34)	28.0 (14)	47.1 (8)
Odd ratio (95% CI)	1.000	2.732 (1.392-5.363)	1.000	0.972 (0.466-2.026)	2.222 (0.792-6.238)
*P *	0.003		0.278		

Weight/fat loss supplements cluster					
Percentage (*n*)	20.9 (28)	5.8 (3)	21.8 (26)	6.0 (3)	11.8 (2)
Odd ratio (95% CI)	1.000	0.232 (0.067-0.799)	1.000	0.228 (0.066-0.793)	0.477 (0.102-2.221)
*P *	0.013		0.035		

Alertness/energy supplements cluster					
Percentage (*n*)	25.4 (34)	9.6 (5)	25.2 (30)	14.0 (7)	11.8 (2)
Odd ratio (95% CI)	1.000	0.313 (0.115-0.851)	1.000	0.483 (0.196-1.187)	0.396 (0.085-1.831)
*P *	0.018		0.163		

Soy and sports bar cluster					
Percentage (*n*)	11.2 (15)	17.3 (9)	15.1 (18)	8.0 (4)	11.8 (2)
Odd ratio (95% CI)	1.000	1.660 (0.677-4.072)	1.000	0.488 (0.156-1.523)	0.748 (0.157-3.554)
*P *	0.264		0.447		

Natural supplements cluster					
Percentage (*n*)	13.4 (18)	63.5 (33)	18.5 (22)	34.0 (17)	70.6 (12)
Odd ratio (95% CI)	1.000	11.193 (5.278-23.737)	1.000	2.271 (1.077-4.789)	10.582 (3.380-33.131)
*P *	<0.001		<0.001		

Protein powder					
Percentage (*n*)	55.2 (74)	0 (0)	43.7 (52)	40.0 (20)	11.8 (2)
Odd ratio (95% CI)	1.000	0.000	1.000	0.859 (0.439-1.682)	0.172 (0.038-0.785)
*P *	<0.001		0.042		

Casein protein					
Percentage (*n*)	3.0 (4)	0 (0)	2.5 (3)	0.0	5.9 (1)
Odd ratio (95% CI)	1.000	0.000	1.000	0.000	2.417 (0.237-24.659)
*P *	0.208		0.316		

Whey protein					
Percentage (*n*)	41.0 (55)	9.6 (5)	31.9 (38)	38.0 (19)	17.6 (3)
Odd ratio (95% CI)	1.000	0.153 (0.057-0.409)	1.000	1.306 (0.656-2.602)	0.457 (0.124-1.685)
*P *	<0.001		0.298		

Antioxidants					
Percentage (*n*)	3.0 (4)	7.7 (4)	1.7 (2)	8.0 (4)	11.8 (2)
Odd ratio (95% CI)	1.000	2.708 (0.651-11.260)	1.000	5.087 (0.901-28.731)	7.800 (1.022-59.529)
*P *	0.156		0.051		

**Table 4:** Reasons for supplement use by gender and age group (*n* = 186).

Reasons for supplement use	Gender		Age group (years)		
	Men	Woman	20–30	30–40	40–50
Disease prevention cluster					
Percentage (*n*)	7.5 (10)	69.2 (36)	17.6 (21)	22.0 (11)	82.4 (14)
Odd ratio (95% CI)	1.000	27.900 (11.655–66.787)	1.000	1.316 (0.581–2.984)	21.778 (5.742–82.594)
*P *	<0.001		<0.001		

Immunity/energy boosting cluster					
Percentage (*n*)	9.7 (13)	17.3 (9)	12.6 (15)	8.0 (4)	17.6 (3)
Odd ratio (95% CI)	1.000	1.948 (0.7778–4.880)	1.000	0.603 (0.190–1.916)	1.486 (0.382–5.785)
*P *	0.149		0.516		

Muscle building cluster					
Percentage (*n*)	85.1 (114)	40.4 (21)	81.5 (97)	62.0 (31)	41.2 (7)
Odd ratio (95% CI)	1.000	0.119 (0.057–0.247)	1.000	0.370 (0.177–0.772)	0.159 (0.054–0.463)
*P *	<0.001		<0.001		

Weight/fat loss cluster					
Percentage (*n*)	50.7 (68)	23.1 (12)	42.0 (50)	52.0 (26)	23.5 (4)
Odd ratio (95% CI)	1.000	0.291 (0.141–0.603)	1.000	1.495 (0.770–2.903)	0.425 (0.131–1.379)
*P *	0.001		0.115		

There are no missing data.

CI: confidence interval.

**Table 5:** Source of supplement information by gender and age group (*n* = 186).

Source of information	Gender		Age group (years)		
	Men	Woman	20–30	30–40	40–50
Medical/paramedical cluster					
Percentage (*n*)	42.5 (57)	88.5 (46)	46.2 (55)	66.0 (33)	88.2 (15)
Odd ratio (95% CI)	1.000	10.357 (4.139–25.912)	1.000	2.259 (1.136–4.491)	8.727 (1.911–39.854)
*P *	<0.001		0.001		

Media cluster					
Percantage (*n*)	55.2 (74)	21.2 (11)	50.4 (60)	38.0 (19)	35.3 (6)
Odd ratio (95% CI)	1.000	0.218 (0.013–0.459)	1.000	0.603 (0.307–1.183)	0.536 (0.186–1.544)
*P *	<0.001		0.223		

Coaches cluster					
Percantage (*n*)	61.2 (82)	5.8 (3)	52.1 (62)	42.0 (21)	11.8 (2)
Odd ratio (95% CI)	1.000	0.039 (0.012–0.131)	1.000	0.666 (0.342–1.297)	0.123 (0.027–0.560)
*P *	<0.001		0.006		

There are no missing data.

CI: confidence interval.
